# Impact of Individual and Environmental Socioeconomic Status on Peritoneal Dialysis Outcomes: A Retrospective Multicenter Cohort Study

**DOI:** 10.1371/journal.pone.0050766

**Published:** 2012-11-30

**Authors:** Rong Xu, Qing-Feng Han, Tong-Ying Zhu, Ye-Ping Ren, Jiang-Hua Chen, Hui-Ping Zhao, Meng-Hua Chen, Jie Dong, Yue Wang, Chuan-Ming Hao, Rui Zhang, Xiao-Hui Zhang, Mei Wang, Na Tian, Hai-Yan Wang

**Affiliations:** 1 Renal Division, Department of Medicine, Peking University First Hospital, Institute of Nephrology, Peking University, Key Laboratory of Renal Disease, Ministry of Health, Key Laboratory of Renal Disease, Ministry of Education, Beijing, China; 2 Department of Nephrology, Peking University Third Hospital, Beijing, China; 3 Department of Nephrology, Huashan Hospital of Fudan University, Shanghai, China; 4 Department of Nephrology, Second Affiliated Hospital of Harbin Medical University, Heilongjiang, China; 5 Kidney Disease Center, The First Affiliated Hospital, College of Medicine, Zhejiang University, Hangzhou, China; 6 Department of Nephrology, Peking University People’s Hospital, Beijing, China; 7 Department of Nephrology, General Hospital of Ningxia Medical University, Ningxia, China; Cardiff University School of Medicine, United Kingdom

## Abstract

**Objectives:**

We aimed to explore the impacts of individual and environmental socioeconomic status (SES) on the outcome of peritoneal dialysis (PD) in regions with significant SES disparity, through a retrospective multicenter cohort in China.

**Methods:**

Overall, 2,171 incident patients from seven PD centers were included. Individual SES was evaluated from yearly household income per person and education level. Environmental SES was represented by regional gross domestic product (GDP) per capita and medical resources. Undeveloped regions were defined as those with regional GDP lower than the median. All-cause and cardiovascular death and initial peritonitis were recorded as outcome events.

**Results:**

Poorer PD patients or those who lived in undeveloped areas were younger and less-educated and bore a heavier burden of medical expenses. They had lower hemoglobin and serum albumin at baseline. Low income independently predicted the highest risks for all-cause or cardiovascular death and initial peritonitis compared with medium and high income. The interaction effect between individual education and regional GDP was determined. In undeveloped regions, patients with an elementary school education or lower were at significantly higher risk for all-cause death but not cardiovascular death or initial peritonitis compared with those who attended high school or had a higher diploma. Regional GDP was not associated with any outcome events.

**Conclusion:**

Low personal income independently influenced all-cause and cardiovascular death, and initial peritonitis in PD patients. Education level predicted all-cause death only for patients in undeveloped regions. For PD patients in these high risk situations, integrated care before dialysis and well-constructed PD training programs might be helpful.

## Introduction

Recently, the China National Survey of Chronic Kidney Disease Working Group reported that the prevalence of chronic kidney disease was 10.8%, close to that in Western countries [1]. It is predicted that end stage renal disease (ESRD) will increase rapidly and become highly prevalent [2]. In China, medical insurance will be extended to cover all ESRD patients as proposed in a government working report in 2012 (http://english.gov.cn/official/2012-03/15/content_2092737.htm). Because peritoneal dialysis (PD) is less expensive [3], has a comparable survival rate [4], [5] and can confer a better quality of life than hemodialysis (HD) [6], [7], a series of healthcare policies have been considered to increase the penetration of PD (http://www.moh.gov.cn/publicfiles/business/htmlfiles/chenz/pldjh/201107/52373.htm). At present, the possibility of popularizing PD treatment for ESRD patients urgently needs to be explored.

As a home-care therapy, PD requires patients to self-monitor and self-manage their treatment [8], [9], [10], abilities that are closely associated with socioeconomic status (SES) [11], [12]. SES has been investigated for its association with the outcomes of treatment in the general population and in patients with chronic kidney disease at the individual [13], [14], [15], [16], [17] and environmental [13], [15], [16], [17], [18] level. A few large-scale multi-center cohort studies have also explored this issue in PD patients and shown inconsistencies in the impact of individual [19], [20], [21], [22], [23] and environmental [21], [22], [24], [25], [26], [27] SES on PD outcome. In China, we have experienced great economic development in the last 20 years, with an increase in gross domestic product (GDP) of around 10% per year, but there are imbalances between regions. Individual income and education levels also vary markedly within regions and probably lead to diverse availability of medical facilities and health-care services. Given this situation, a specific model is needed to determine whether variations in individual or environmental SES influence PD outcome. Moreover, the interaction between individual and environmental SES on PD outcome, which has never been studied in a dialysis population, should be explored.

**Table 1:** Baseline characteristics according to individual income.

		Individual income			P
	Total	Low(<¥20,000, i.e., <$3160)	Medium(¥20,000-¥40,000, i.e., $3160∼$6320)	High(>¥40,000, i.e., >$6320)	
Proportion (%)	100	36.5	39.8	23.7	----
Age>65yrs (%)	41.5	33.0	39.8	46.0	<0.001
Male (%)	49.5	46.8	51.6	51.1	0.10
Body mass index (kg/m^2^)	22.9±3.6	22.8±3.7	23.1±3.6	23.0±3.5	0.27
DM (%)	37.6	34.7	39.7	39.9	0.13
CGN (%)	34.9	37.6	34.4	30.7	0.18
CVD (%)	40.9%	37.5	44.9	40.9	0.01
High school or above (%)	44.3	33.9	45.0	67.7	<0.001
Proportion of individual income used for medicalexpenses *>50% (%)	48.4	76.6	33.5	17.2	<0.001
Rural residence (%)	19.5	27.3	17.2	6.1	<0.001
Live alone (%)	3.1	3.0	3.3	2.6	0.17
Distance from hospital (KM)	20(8∼80)	32(10∼150)	20(7∼79)	12(6∼32)	<0.001
Frequent visitors ** (%)	85.4	82.4	88.8	88.3	<0.001
Live in undeveloped region (%)	55.9	64.7	55.2	38.6	<0.001
Hemoglobin (g/L)	103.9±19.2	100.8±19.0	106.8±18.9	106.5±18.9	<0.001
Serum albumin (g/L)	35.5±5.3	34.9±5.5	36.0±5.2	36.3±4.7	<0.001
RRF (ml/min)	4.67(3.52–6.09)	4.67(3.41–6.15)	4.70(3.57–6.06)	4.58(3.70–6.10)	0.74
Baseline dialysis dosage (ml)	5874±2009	5809±1987	5947±2001	5868±2095	0.49
Total kt/v	1.96(1.65–2.37)	1.95(1.61–2.38)	1.95(1.65–2.38)	1.97(1.70–2.33)	0.90

DM: diabetes mellitus; CGN: chronic glomerulonephritis; CVD: cardiovascular disease; RRF: residual renal function. *Proportion of individual income used for medical expenses was calculated as the percentage of yearly household income per person used each year for yearly self-paid medical expenses. **Frequent visitors were those who visited a doctor at least once every 3 months.

This multicenter large-scale retrospective cohort study will be helpful in providing evidence for clinicians deciding whether PD is suitable for individuals with differing SES, and for health policy-makers exploring potential strategies for establishing PD programs in regions with varying SES in China and other developing countries.

## Methods

### Center Enrollment

Centers with professional PD doctors and nurses and well-developed databases maintained for least 3 years, recording baseline characteristics and follow-up data for Chinese patient every 1∼3 months, participated in this study voluntarily. Nine centers were qualified, and seven of these, accouting for about 70% of all incident patients attending the nine centers, agreed to participate. The included PD centers were located in five different provinces and four geographical regions (north, northeast, northwest, and east) of China. Data from each center were collected within a strict quality control framework and further inspected and optimized to ensure the integrity and accuracy of the database. All study investigators and staff members completed a training program that taught them the methods and processes of the study. A manual of detailed instructions for data collection was distributed. The ethics committee of Peking University First Hospital approved the study.

**Table 2:** Baseline characteristics according to regional GDP.

	Regional GDP		P
	undeveloped(GDP per capital<¥95000 i.e $15009)	Developed(GDP per capital≥¥95000 i.e $15009)	
Age>65yrs (%)	30.6	53.2	<0.001
Male %	50.1%	47.3%	0.21
Body mass index (kg/m^2^)	22.9±3.6	23.2±3.6	0.08
DM (%)	34.1	45.6	<0.001
CGN (%)	37.7	26.8	<0.001
CVD (%)	40.2	43.2	0.19
Low individual income (%)	48.3%	33.7%	<0.001
High school or above (%)	38.7	52.9	<0.001
Proportion of individual income used for medical expenses*>50% (%)	57.6	30.5	<0.001
Rural residence (%)	28.9	2.0	<0.001
Live alone (%)	2.9	3.4	0.76
Distance to hospital (KM)	40(20∼130)	6.8(3.7∼11.6)	<0.001
Frequent visitors ** (%)	85.0	92.2	<0.001
Hemoglobin (g/L)	103.2±19.1	106.3±18.4	<0.001
Serum albumin (g/L)	35.8±5.5	35.2±4.9	0.02
RRF (ml/min)	4.70(3.52–6.05)	4.61(3.52–6.05)	0.91
Baseline dialysis dosage (ml)	5900±1939	5909±2104	0.94
Total kt/v	1.94(1.63–2.37)	1.98(1.67–2.37)	0.54
Registered doctors per thousand inhabitants	4.5±2.3	6.2±1.7	<0.001
Available hospital beds per thousand inhabitants	7.7±5.1	7.8±3.0	0.49

DM: diabetes mellitus; CGN: chronic glomerulonephritis; CVD: cardiovascular disease; RRF: residual renal function.

* Proportion of individual income used for medical expenses was calculated as the percentage of yearly household income per person used each year for yearly self-paid medical expenses.

** Frequent visitors were those who visited a doctor at least once every 3 months.

**Table 3:** Detailed clinical outcome of the study population.

Peritoneal dialysis outcome	n(%)
Death	553
cardiovascular disease	210(38.0%)
* myocardial infarction*	*46*
* congestive heart failure*	*41*
* cerebral bleeding*	*30*
* cerebral infarction*	*29*
* arrhythmia*	*10*
* peripheral arterial disease*	*3*
* Sudden death*	*20*
* undefined causes*	*31*
infection	140(25.3%)
malignancy	66(11.9%)
gastrointestinal bleeding	23(4.2%)
malnutrition	27(4.9%)
miscellaneous	25(4.5%)
undefined	62(2.9%)
**Transfer to hemodialysis**	168
peritoneal dialysis related infection	77(45.8%)
leakage	13(7.7%)
hernia	5(3.0%)
catheter disposition	4(2.4%)
ultrafiltration failure	9(5.4%)
severe congestive heart failure	3(1.8%)
dialysis inadequacy	15(8.9%)
miscellaneous	30(17.9%)
undefined	12(7.1%)

**Table 4:** Predicting the roles of individual income and education level in all-cause death, cardiovascular death, and initial peritonitis.

	All cause death		Cardiovascular death		Initial peritonitis	
	adjusted HR* (95% CI)	*P*	adjusted HR* (95% CI)	*P*	adjusted HR* (95% CI)	*P*
**Individual income group**						
* Low (<¥20,000, i.e., <$3160)*	Ref		Ref		Ref	
* Medium (¥20,000-¥40,000, i.e., $3160∼$6320)*	0.62(0.48–0.79)	<0.001	0.60(0.41–0.90)	0.012	0.91(0.71–1.16)	0.44
* High (>¥40,000, i.e., >$6320)*	0.44(0.33–0.61)	<0.001	0.47(0.29–0.76)	0.002	0.69(0.50–0.94)	0.02
**Education level**						
* Elementary or lower*	Ref		Ref		Ref	
* Middle school*	0.76(0.58–1.01)	0.06	0.81(0.53–1.26)	0.36	0.97(0.72–1.30)	0.84
* High school*	0.78(0.57–1.06)	0.12	0.79(0.49–1.28)	0.34	0.95(0.69–1.32)	0.77
* Higher than high school*	0.68(0.50–0.93)	0.02	0.54(0.32–0.91)	0.02	0.84(0.60–1.18)	0.31
Regional socioeconomic status						
* Undeveloped region (GDP per capita* *<¥95,000 i.e $15,009)*	Ref		Ref		Ref	
* Developed region (GDP per capita* *≥¥95,000 i.e $15,009)*	0.88(0.70–1.12)	0.30	0.73(0.51–1.04)	0.09	1.11(0.88–1.42)	0.38

* Adjusted for confounders such as age, gender, body mass index, diabetes, cardiovascular disease, hemoglobin, serum albumin, and residual renal function, and stratified by center size.

### Subject Selection

All incident patients receiving chronic PD between the date of intact database creation and August 2011 were enrolled into this study. After starting PD, each patient signed informed consent agreeing to the use of their demographic and laboratory data in future studies. All subjects began the PD program within 1 month after catheter implantation and were given lactate-buffered glucose dialysate with a twin-bag connection system (Baxter Healthcare, Guangzhou, China). Patients who had been on PD for less than 3 months were excluded, as in previous studies [20], [21].

**Figure 1:**
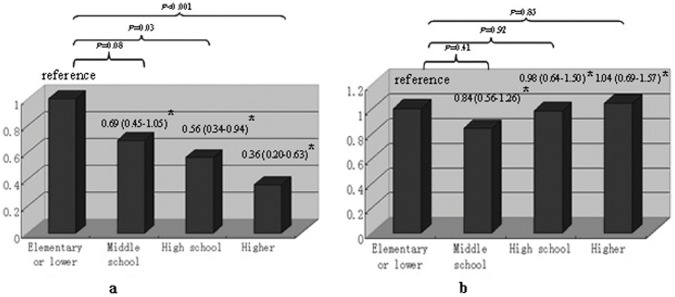
Subgroup analysis of predicted role of education level in all-cause death according to regional economic status. *Adjusted hazard ratio with 95% confidence interval (adjusted for confounders such as age, gender, body mass index, diabetes, cardiovascular disease, hemoglobin, serum albumin, and residual renal function, and stratified by center size) **Undeveloped region: gross domestic product (GDP) per capita <¥95,000 ($15,009); developed region: GDP per capita≥¥95,000 ($15,009).

### Data Collection

Demographic and clinical data including age, gender, body mass index (BMI), primary renal disease, history of cardiovascular disease (CVD), and presence of diabetes mellitus (DM) were collected at baseline. CVD was recorded if one of the following conditions was present: angina, class III/IV congestive heart failure (New York Heart Association), transient ischemic attack, history of myocardial infarction or cerebrovascular accident, or peripheral arterial disease [28]. Baseline biochemistry data including hemoglobin and serum albumin were calculated as the mean of measurements made during the first 3 months. Dialysis adequacy and residual renal function (RRF) were measured during the first 6 months. RRF was defined as the mean of residual creatinine clearance and residual urea clearance. Dialysis adequacy was determined from the total Kt/V and total creatinine clearance. Center size was also recorded according to the number of enrolled patients from each center.

SES data were collected for each patient. Individual income level was defined as the yearly household income per person and was divided into low (<¥20,000, <$3160,), medium (¥20,000–40,000, $3160–6320) and high (>¥40,000, >$6320); because most of the subjects were from urban areas, these groups were defined according to the average income for the urban population in 2011, obtained from the Bureau of Statistics (http://www.bjstats.gov.cn/nj/main/2011-tjnj/index.htm). The exchange rate of the US dollar ($) to the Chinese Yuan (¥) was set at 6.3293 on November 1, 2011. Education levels were recorded according to diplomas obtained based on school level, that is, elementary school or lower, middle school, high school, or above high school. Other individual SES data included the proportion of individual income used for medical expenses (the percentage of yearly household income per person spent each year on self-paid medical expenses), occupation, rural or urban residence, living alone or not, travel distance from the PD center, and frequency of visits to the PD center. A frequent visitor was defined as someone who saw a doctor at least once every 3 months. Regional SES included regional GDP per capita and regional medical resources such as number of registered doctors and available hospital beds per inhabitant. Data were obtained from the Bureau of Statistics in each province; Beijing data were downloaded from http://www.bjstats.gov.cn/nj/main/2011-tjnj/index.htm. Regions were divided into undeveloped regions and developed according to the median of regional GDP per capita (¥95000, $15009).

### Definition of Outcome Events

The Primary outcome was all-cause death or cardiovascular death. Cardiovascular death was defined as death due to myocardial infarction, congestive heart failure, cerebral bleeding, cerebral infarction, arrhythmia, or peripheral arterial disease, or sudden death. The secondary outcome was initial peritonitis, which was diagnosed according to International Society for Peritoneal Dialysis 2010 guidelines [29]. In all analyses, we censored follow-up at transferring to HD, loss to follow-up, renal transplantation, or the end of the study (November 1, 2011).

### Statistical Analysis

Continuous data were presented as means with standard deviation except for distance to PD center, RRF, and total Kt/V, which were presented as the median (interquartile range) because of high skew. Categorical variables were presented as proportions. Relevant characteristics were compared between different individual income groups and between different education groups, respectively. Patient data were compared using the t-test or the analysis of variance F-test for normally distributed continuous variables, the chi-square test for categorical variables, or the Mann–Whitney U test for skewed continuous variables.

To determine predictors of outcome events, univariable Cox regression models were first constructed to explore individually the potential risk factors for all-cause death including demographic and bioclinical data, individual income, education level, and regional GDP per capita. Risk factors identified from univariable Cox regression models were included in multivariable analyses with all-cause death, CVD death and initial peritonitis as events. In multivariable analysis, stratified Cox regression models with center size as the stratified factor were employed to adjust for center effects. The center effect was reflected not only in disparity of center size (ranging from 78 patients to 815 patients), which has been demonstrated to be an independent predictor of PD outcome [30], [31], [32], but also in differences in practice patterns and biochemical assays between centers, which differed from the regional effect. The regional effect was defined by regional GDP per capita and reflected differences in SES between regions. To determine whether individual income or education level interacted with regional GDP per capita, we explored their two-way interaction effects on the likelihood of patient survival and peritonitis-free survival. When an interactive effect was observed, subgroup analysis was performed.

We reported the multivariable adjusted hazard ratios (HRs) with 95% confidence interval (CI). All probabilities were two-tailed, and the level of significance was set at 0.05. Statistical analyses were performed using SPSS for Windows software version 13.0 (SPSS Inc., Chicago, IL).

## Results

### Baseline Characteristics

Data from 2409 patients were collected. One hundred and forty-five patients were excluded because they began the PD program before the date of intact database creation or more than 1 month after catheter implantation. Another 93 patients who had been receiving PD for less than 3 months were also excluded. The included 2171 patients had a mean age of 58.0±15.5 years and BMI 22.9±3.6 kg/m^2^; 37.6% were diabetic and CVD was present in 40.9% of the subjects at baseline. Chronic glomerulonephritis (CGN) was the most common cause of ESRD (34.9%), followed by diabetic nephropathy and hypertensive nephropathy. The proportions of patients with low, median and high income were 36.5%, 39.8% and 23.7% respectively; 44.3% had a diploma from high school or above. There was significant regional economic disparity across the whole cohort, with GDP per capita ranging from the 10^th^ percentile at ¥24,768 ($3913) to the 90^th^ percentile at ¥175,495 ($27,722). Undeveloped regions were areas with GDP per capita lower than the median (¥95,000, $15,009) and had fewer registered doctors than developed regions but comparable numbers of available hospital beds per thousand inhabitants (4.5±2.3 vs 6.2±1.7, P<0.001 for doctors; 7.7±5.1 vs 7.8±3.0, P = 0.49 for beds).

### Socioeconomic and Clinic Characteristics

Patients with low individual income were less likely to be elderly (33.0%) and have a history of CVD (37.5%). The lowest individual incomes were associated with the lowest education level and highest proportion of individual income used for medical expenses. The poorest patients were also more likely to live in rural areas and undeveloped regions at the greatest distances from hospital. Accordingly, the percentage of frequent visitors was lowest in the low individual income group. At baseline, hemoglobin and serum albumin were lowest in the low individual income group. However, gender, BMI, prevalence of DM and CGN, living alone, RRF, baseline dialysis dose, and total Kt/V did not differ significantly between income groups (**Table 1**).

Patients who lived in undeveloped regions were significantly younger, poorer, and less educated and lived further from hospital. There were more cases of diabetes and fewer cases of CGN in this group. Hemoglobin and serum albumin were lower, but RRF and dialysis dose were comparable to those in developed regions. Undeveloped regions had fewer registered doctors per thousand inhabitants than developed regions. These data are shown in **Table 2**.

### Follow-up and Outcome

The median follow-up time was 27.7(15.5–45) months. As shown in Table 3, among 553 patients who died, 210 deaths (38%) were due to CVD and 140 (25.3%) to infection; other causes were malignancy, gastrointestinal bleeding, malnutrition, miscellaneous, and undefined. Of the 210 patients who died from CVD, the leading cause was myocardial infarction (46 cases, 21.9%); other causes were congestive heart failure, cerebral bleeding, cerebral infarction, arrhythmia, peripheral arterial disease, sudden death, and undefined. One hundred and sixty-eight patients were transferred to HD, most due to PD-associated infection (77 cases, 45.8%); other reasons were leakage, hernia, catheter disposition, ultrafiltration failure, severe congestive heart failure, inadequacy of dialysis, miscellaneous, and undefined.

The time to first-episode peritonitis was 23.7 (11.9–40.4) months. Four hundred and fifty-five episodes of initial peritonitis occurred during the study period; 160 episodes (35.2%) were due to Gram-positive organisms, 94 (20.7%) to Gram-negative organisms, and six (1.3%) to fungi; 12 (2.6%) were polymicrobial, 130 (28.6%) were culture negative and 53 had no culture result (11.6%).

### Association between SES and Outcome

On univariable Cox regression analysis, individual income and education level but not regional GDP per capita were significantly associated with all-cause death. The predicted role of these factors were further explored by multivariable Cox regression analysis. Age, BMI, DM, CVD, hemoglobin, serum albumin, RRF, and center size were also found to be significantly associated with all-cause death on univariable Cox regression analysis. After stratification by center and controlling for all of these confounders and gender, compared with the low income group, the adjusted HRs for all-cause death in the medium and high income group were 0.62 (95%CI 0.48–0.79, P<0.001) and 0.44 (95%CI 0.33–0.61, P<0.001) respectively. Medium income and high income were also associated with significantly lower risks for cardiovascular death, with adjusted HRs of 0.60 (95%CI 0.41–0.90 P = 0.012) and 0.47 (95%CI 0.29–0.76, P = 0.002) respectively. The high income group, but not the medium income group had a significantly lower risk for initial peritonitis, with an adjusted HR of 0.69 (95%CI 0.50–0.94, P = 0.02) compared with the low income group (**Table 4**).

Compared with patients with an elementary school education or lower, patients with higher education levels did not show a constant trend toward lower risk for all-cause death. Only a diploma above the high school level predicted significantly lower risk for all-cause death, with an adjusted HR of 0.68 (95%CI 0.50–0.93, *P* = 0.02), and for cardiovascular death, with an adjusted HR of 0.54 (95%CI 0.32–0.91, *P* = 0.02). Otherwise, education level had no effect on cardiovascular death or initial peritonitis (**Table 4**).

Compared with patients living in undeveloped regions, patients in developed regions had similar risk of cardiovascular or all-cause death and initial peritonitis, even after controlling for all of above mentioned confounders such as age, proportion of patients with diabetes, serum albumin, and so on (**Table 4**).

In addition, we explored the interactive effect between individual income, education level and regional GDP per capita. We found that individual education but not income level had a significant interactive effect with regional GDP per capita on all-cause death (adjusted HR 0.79, 95% CI 0.65–0.97, P = 0.024), and this influence was further explored after stratifying the patients according to regional development. In undeveloped regions, an education level of high school or above predicted a significantly lower risk for all-cause death compared with elementary school or lower, with adjusted HRs of 0.56 (95% CI 0.34–0.94, P = 0.03) and 0.36 (95% CI 0.20–0.63, P<0.001), respectively. In developed regions, education level did not predict all-cause death (**Fig. 1**). No interaction effects were observed between individual income or education and regional GDP in terms of cardiovascular death and initial peritonitis.

## Discussion

From this first large-scale multi-center cohort of incident Chinese PD patients, we found that individual rather than regional income independently predicted all-cause death, cardiovascular death, and initial peritonitis. Education level was only significantly associated with all-cause death in undeveloped regions but not in developed regions. An interaction effect of individual education level and environmental SES is reported for the first time.

Consistent with former studies [19], [33], [34], [35], lower individual income emerged as an independently significant risk indicator for death and initial peritonitis. Although poorer patients were younger and had less comorbidity, they bore a heavier burden of medical expenses and had access to fewer medical resources, which strongly supports increasing the personal medical coverage rate for PD patients in China. Of note, poorer patients were prone to be anemic and malnourished at baseline, probably due to inadequate health care and late referral to nephrologists before the development of ESRD. Given that the standard chronic kidney disease program is helpful in retarding progression to ESRD and improving complications such as anemia, malnutrition and bone mineral disease [36], [37], the timing of and strategies used for medical support before dialysis in China need to be urgently investigated. We note that effective medical support aids the establishment of successful PD programs for disadvantaged minorities [38], [39].

By contrast, no impact of regional GDP per capita on PD outcome was observed, the opposite of previous findings showing an independently negative effect of regional SES on outcome in chronic disease [15], [16], [17]. One potential reason for our finding is that, in the undeveloped regions in our study, the patients were younger, there were fewer cases of diabetes, and baseline serum albumin levels were higher than in the developed regions. Given that age, diabetes and serum albumin have been recognized as the strongest predictors by most studies [40], [41], [42], these favorable individual factors in our study possibly offset the disadvantages of regional SES. A similar finding was obtained in a study from the USA [24], in which the risk of technique failure was significantly lower in remote-dwelling patients than in those living closer to the hospital, because the former were younger and had fewer complications from diabetes. However, no association between regional SES and cardiovascular or all-cause death or initial peritonitis was observed, even after adjustment for age, proportion of patients with diabetes, serum albumin, and so on. We hypothesize that unknown confounding factors related to regional SES are also associated with PD outcome. In addition, only baseline characteristics, and not the change trend in clinical variables, were analyzed for the prediction of outcome during follow-up. This choice may have influenced the reliability of our results.

Our analysis indicates that education level had no effect on risk of initial peritonitis. This result is consistent with the analysis of a US regional ESRD registry in which 1595 new PD patients were observed over 2 years [23], but is contrary to recently published data from Brazil and Canada [21], [22]. One possible explanation is that our selected centers had professional PD doctors/nurses and well-constructed training programs. Patients and their home-care helpers were often trained simultaneously, which probably led to stronger family support [43]. Whether better compliance in Asian people [44], [45] plays a role in this phenomemon is unclear. However, lower education level still significantly predicted death in undeveloped regions. The causes for this finding are unknown, but it has been shown that PD networks linking developed and developing units might be a means of improving the quality of therapy [46]. Whether the establishment of a standardized PD program in undeveloped regions will benefit less-educated patients needs to be investigated. More trials are also needed to explore new strategies for improving the efficacy of training for less-educated patients.

Our large-scale multicenter PD cohort gave us a valuable opportunity to explore the association of SES and outcome at both the individual and the environmental level. Our results will be helpful for PD clinicians and health policy-makers in generating appropriate strategies to improve the use of PD in developing countries such as China. Compared with former studies, this study has the advantage of more detailed information on individual SES. The enrollment of representative centers in a rapidly developing country with huge diversity in regional economic development is also a merit. The interactive effect between individual and regional SES has never been investigated in a dialysis population.

This study has some limitations. First, based on estimates of the size of the dialysis population (no registry data available as yet), only about 10% of PD patients in China were enrolled in the study and thus we cannot confirm the generalizability of our results. However, all incident patients were enrolled from ‘core’ PD centers of medical school-affiliated hospitals and came from provinces and counties with varied level of SES and penetration rate of PD therapy. Therefore, it is reasonable to conclude that the range of SES of our study population reflects the overall situation to a certain degree. A national dialysis registry has been initiated recently, so more representative data will be obtained in future years. We Hope our study will provide useful cues for the analysis of national data in the future. Second, individual SES is a general index and many SES-related physiological and non-physiological factors were not measured. We cannot verify whether worse nutritional status, deficient pre-dialysis care, less access to standardized training courses, or poor compliance contributed to the worse outcome observed in poorer patients. Regional SES-related information such as local hygiene status, availability of medical services and social support systems was not assessed in detail. Third, we should be aware of the possibility of ascertainment bias (4.1% of eligible patients were not included), vintage bias (different centers created their databases at different times), and residual confounding and recall bias because of the retrospective nature of this study. Furthermore, an observational study cannot demonstrate cause–effect relationships.

In conclusion, our findings strongly support the present health-care strategies implemented by the Chinese government to improve the medical coverage rate for ESRD patients. Our data also suggest that, under the present training program, the risk for peritonitis in less-educated patients is comparable with that in patients with higher diplomas. A series of strategies should be applied to improve the quality of treatment for poorer patients and less-educated patients in undeveloped region. Constructing an integrated care system for chronic kidney disease patients to prevent various complications and developing PD networks to standardize training programs may be potential approaches to improving the quality of therapy.
